# Walk with ease for knee osteoarthritis: A cost-effectiveness analysis

**DOI:** 10.1016/j.ocarto.2023.100368

**Published:** 2023-05-10

**Authors:** Zoe E. Zimmerman, Rebecca J. Cleveland, Aleksandra M. Kostic, Valia P. Leifer, Serena E. Weisner, Kelli D. Allen, Yvonne M. Golightly, Heather Welch, Melissa Dale, Stephen P. Messier, David J. Hunter, Jeffrey N. Katz, Leigh F. Callahan, Elena Losina

**Affiliations:** a Policy and Innovation EValuation in Orthopaedic Treatments (PIVOT) Center, Department of Orthopaedic Surgery, Brigham and Women's Hospital, Boston, MA, USA; b Orthopaedic and Arthritis Center for Outcomes Research (OrACORe), Department of Orthopaedic Surgery, Brigham and Women's Hospital, Boston, MA, USA; c Thurston Arthritis Research Center; Department of Medicine, University of North Carolina at Chapel Hill, Chapel Hill, NC, USA; d Thurston Arthritis Research Center; Osteoarthritis Action Alliance, University of North Carolina, Chapel Hill, NC, USA; e Durham VA Health Care System, Durham, USA; f College of Allied Health Professions, University of Nebraska Medical Center, Omaha, NE, USA; g Montana Department of Public Health and Human Services, Helena, MT, USA; h J.B. Snow Biomechanics Laboratory, Department of Health and Exercise Science, Wake Forest University, Winston-Salem, NC, USA; i Sydney Musculoskeletal Health, Kolling Institute, University of Sydney and Rheumatology Department, Royal North Shore Hospital, Sydney, Australia; j Harvard Medical School, Boston, MA, USA; k Division of Rheumatology, Inflammation and Immunity, Brigham and Women's Hospital, Boston, MA, USA; l Thurston Arthritis Research Center Departments of Medicine and Orthopaedics, Osteoarthritis Action Alliance, Dept. of Epidemiology, Gillings School of Global Public Health, University of North Carolina, Chapel Hill, USA; m Department of Epidemiology, Harvard T.H. Chan School of Public Health, Boston, MA, USA; n Department of Biostatistics, Boston University School of Public Health, Boston, MA, USA; o University of North Carolina at Chapel Hill, Chapel Hill, NC, USA; p Thurston Arthritis Research Center and Osteoarthritis Action Alliance, University of North Carolina, Chapel Hill, NC, USA

**Keywords:** Knee osteoarthritis, Physical activity, Exercise, Cost-effectiveness

## Abstract

**Objective:**

The Walk With Ease (WWE) program was developed by the Arthritis Foundation to help people with arthritis learn to exercise safely and improve arthritis symptoms. We sought to establish the value of the WWE program.

**Methods:**

We used the Osteoarthritis Policy (OAPol) Model, a widely published and validated computer simulation of knee osteoarthritis (OA), to assess the cost-effectiveness of WWE in knee OA. We derived model inputs using data from a workplace wellness initiative in Montana that offered WWE to state employees. Our primary outcomes were quality-adjusted life years (QALYs) and costs over a 2-year period, which we used to calculate the incremental cost-effectiveness ratio (ICER). The base case analysis was restricted to subjects who were inactive or insufficiently active (<180 ​min/week of PA) at baseline. We performed scenario and probabilistic sensitivity analyses to determine the impact of uncertainty in model parameters on our results.

**Results:**

In the base case analysis, adding WWE to usual care resulted in an ICER of $47,900/QALY. When the program was offered without preselection by baseline activity level, the ICER for WWE ​+ ​usual care was estimated at $83,400/QALY. Results of the probabilistic sensitivity analysis indicated that WWE offered to inactive or insufficiently active individuals has a 52% chance of having an ICER <$50,000/QALY.

**Conclusion:**

The WWE program offers good value for inactive/insufficiently active individuals. Payers may consider including such a program to increase physical activity in individuals with knee OA.

## 1 Introduction

Physical activity (PA) positively impacts health by improving quality of life (QoL), delaying functional decline, and preventing comorbidities such as cardiovascular disease and diabetes mellitus [[1], [2], [3]]. Moreover, people who meet the 2018 Physical Activity Guidelines for Americans, 2nd edition (≥150 ​min/week of moderate-to-vigorous PA [4]) have lower total healthcare expenditures than those who do not [5,6]. Despite the numerous benefits associated with PA, many Americans, including 44% of men and 22% of women with knee osteoarthritis (OA), do not meet the guidelines [7]. Further, a recent study found that 30% of adults with arthritis report no physical activity in the last 30 days [8]. Exercise may be particularly beneficial for those with knee OA, as it can improve knee pain and function [9]. Physical inactivity in this population has been shown to result in a loss of 7.5 million total quality-adjusted life years (QALYs) (0.55 QALYs/person) [10]. These data emphasize the need for interventions to increase PA levels among individuals with knee OA.

Physical activity programs, while efficacious for increasing participant activity levels and reducing functional impairments and symptoms, have not been widely implemented in the management of knee OA [11]. Cost-effectiveness analyses can help determine the potential value of offering these interventions to specified populations. A recent analysis found that an intensive diet and exercise program for persons with knee OA was cost-effective relative to usual care at a willingness-to-pay (WTP) threshold (how much policy-makers would be willing to pay for one year of perfect health) of $50,000 (USD) [12]. A New Zealand study also found that an exercise therapy program was cost-effective relative to usual care at WTP thresholds ≥ the per capita gross domestic product [13]. An article from Sevick et al. reported that both aerobic and resistance training exercise programs were cost-saving compared to an education control for seniors with knee OA [14]. These analyses indicate that adding an exercise program to usual care for knee OA is either cost-saving or cost-effective in comparison with usual care alone; however, the programs evaluated have generally required supervision by a physiotherapist and were delivered over a timeframe of months or years [[12], [13], [14]]. Physical therapy programs may not be accessible to patients because the cost is too high (particularly if patients are un- or underinsured), programs are too time-consuming, or patients have difficulty finding adequate and reliable transportation [15,16]. There is a need for analyses to determine the cost-effectiveness of various types of PA programs, including those that are less costly, shorter-term, and that either do not require physiotherapist involvement or generally require little staff involvement.

The Walk With Ease (WWE) program was established by the Arthritis Foundation to help participants develop a walking plan, learn to exercise safely and comfortably, set PA goals, and reduce arthritis symptoms [17]. The program is 6 weeks in duration and can be completed in an instructor-led group setting or self-directed using a workbook. The self-directed program was added to an ongoing workplace wellness program for Montana state employees in 2015 [18]. At the time of the current analysis, data for implementation of the WWE program are only available for the study in Montana. The WWE program has a relatively low administrative cost: participants only need a guidebook, a place to walk, and appropriate shoes. Data from the implementation of this program in Montana suggest that it is effective in increasing PA short-term in a large sample of employees [18]. In the current study, we conduct a cost-effectiveness analysis to determine the value of the WWE program. To our knowledge, no prior cost-effectiveness analyses have been carried out for a short-term, low-cost PA program such as WWE. We also sought to determine whether investing additional resources into the program to increase participant adherence would be cost-effective.

## 2 Materials and methods

### 2.1 Analytic overview

We used the Osteoarthritis Policy (OAPol) Model, a widely published and validated computer simulation model of knee OA [[19], [20], [21], [22]], to assess the cost-effectiveness of adding WWE to the model's usual care regimen for knee OA (NSAIDs/physical therapy and intraarticular corticosteroid injections). The OAPol model and this study are approved by the Mass General Brigham IRB (Protocol #2006P001290). Our primary outcome was the incremental cost-effectiveness ratio (ICER), calculated as the difference in cost divided by the difference in quality-adjusted life years (QALYs) between usual care and WWE ​+ ​usual care over a 2-year timeframe.

We categorized subjects with knee OA into three groups based on their PA level at baseline: inactive (0–30 ​min of PA/week), insufficiently active (31–179 ​min of PA/week), and active (≥180 ​min of PA/week). These cutoffs were based on the self-reported data collected in the Montana study (see Technical Appendix) [18].

Costs and QALYs were discounted at 3% annually, and costs are reported in 2020 USD. We conducted analyses from the health care sector perspective (only considering healthcare costs and not considering costs due to lost productivity, cost of time spent waiting for doctor's appointments, etc.)

#### 2.1.1 The OAPol model structure

OAPol is a Monte-Carlo state transition simulation used to portray the natural history and treatment of knee OA [[23], [24], [25]]. User-defined distributions determine demographic (e.g., sex and age) and clinical characteristics (e.g., knee OA severity, pain, and PA level) of the cohort at baseline. The model can run on monthly or annual cycles; we used monthly cycles for this analysis. Each model cycle, or month, subjects are assigned a QoL utility value between 0 and 1 (0 being death and 1 being perfect health) [26] and accrue costs due to OA treatment and non-OA-related healthcare [27,28]. Pain due to OA, obesity, and comorbidities such as cancer and cardiovascular disease can all decrease a subject's QoL utility value. Transition probabilities govern how subjects move through health states defined by obesity, knee pain level, structural OA severity, and presence of comorbidities. Pain levels are defined by five groups: no pain (WOMAC pain ​= ​0), lowest pain (0 ​< ​WOMAC pain ≤15), low-moderate pain (15 ​< ​WOMAC pain ≤40), moderate pain (40 ​< ​WOMAC pain ≤70), and severe pain (70 ​< ​WOMAC pain ≤100). The model captures several comorbidities prevalent among persons with knee OA (type 2 diabetes mellitus, cancer, cardiovascular disease, chronic obstructive pulmonary disease, and other musculoskeletal diseases other than OA).

Background medical costs and QoL utility values are stratified by number of comorbidities, obesity class, age, and PA level. QoL is additionally stratified by pain level. Interventions that result in a change in any one of these parameters (e.g., pharmacological interventions that decrease pain or exercise interventions that increase PA) therefore, have the potential to change costs accrued and QALYs.

In OAPol, biomedical interventions are characterized by their efficacy, duration (in cycles), cost (start-up and per cycle), and risk of complications. We modeled WWE as an intervention affecting PA levels. The efficacy of a PA program in OAPol is defined as its ability to move participants from a less active PA group to a more active PA group and to decrease pain. Over time, subjects can retain their improved levels of PA and/or pain or regress to their initial pain level and PA group. Those who are insufficiently active or active have lower healthcare expenditures and higher QoL utility values in each cycle than those who are inactive. Moving from a lower to a higher PA level increases QoL, independent of any benefits PA may have on knee pain.

OAPol aggregates results from large numbers of simulations to produce stable, population-level estimates of outcomes. Model output includes average discounted QALYs and costs for subjects over the time frame of the analysis (two years for the analysis presented in this report).

### 2.2 Assumptions

Our analysis is based on several assumptions.

1. WWE is not associated with adverse events, as none were reported in the Montana study [18].
2. WWE does not lead to a reduction in pain, as the average pain for the cohort with arthritis in the Montana study did not change significantly from baseline to either of the follow up timepoints [18]. Increasing PA results in QoL and cost improvements, independent of the impact PA has on pain.
3. Overall, BMI does not impact the effect of WWE on PA (no BMI data were available for Montana study participants).
4. To be conservative, we assumed that those who did not report data at the 6-month follow-up returned to their background activity levels.
5. Those who did not increase their activity group by the end of month one stay in their initial PA group for the remainder of the simulation.

### 2.3 Input parameters

#### 2.3.1 Cohort characteristics

We modeled two cohorts: 1) restricted cohort, including those who were inactive or had insufficient PA levels at baseline and 2) unrestricted cohort, including those with any PA level at baseline. The Montana study offered WWE to all employees and individuals of all activity levels participated. However, since those who meet the PA recommendations at baseline already experience the maximum QoL and cost benefits due to their activity level, their QoL cannot be increased and their cost cannot be decreased further by participating in the WWE program. We decided to model separately a cohort of subjects who all had the potential to increase their PA level and experience QoL and cost improvements. Payers would likely want to target this group of inactive/insufficiently active individuals for the WWE program for maximal cost-effectiveness.

We derived cohort characteristics from the subset of Montana study participants that reported having been told by a health professional that they have any type of arthritis. The mean (SD) age of the cohort was 57.5 (10.3), 73% of subjects were female, and 100% of men and 99.7% of women were white and non-Hispanic. We derived prevalence and incidence of comorbidities using the National Health and Nutrition Examination Survey (NHANES) 2017–2018 and relative mortality risks using NHANES and Centers for Disease Control and Prevention Life Tables, 2017–2018 [29].

Osteoarthritis pain and KL grade: Participant-reported pain severity in the Montana study was assigned using the WOMAC pain subscale. Of the subjects, 84% had pain >0, and the mean (SD) pain of the entire cohort at baseline was 38.2 (21.8) (0–100 scale with 100 ​= ​most severe pain). All the subjects (100%) have KL grade 2 ​at baseline.

##### 2.3.1.1 PA levels

The PA group distribution at baseline was derived from self-reported data from the Montana study [18]. Relative to inactive subjects, those who are insufficiently active and active have lower annual costs (by $351 and $799, respectively) and higher QoL (by 0.019 and 0.037 QALY, respectively) (Table 1). Subjects who are insufficiently active or active also have a lower prevalence and incidence of cardiovascular disease, diabetes mellitus, and cancer than those who are inactive (see Technical Appendix for values stratified by activity group).

**Table 1:** Key model inputs.

Population Characteristics	Mean (SD^a^)	Derived from sources
Age (years)	57.5 (10.3)	18
Female	73%	18
Race/Ethnicity		
White non-Hispanic	99.7%	18
White Hispanic	0.3%	18
BMI (kg/m^2^)		
Restricted Cohort	28 (6)	29
Unrestricted Cohort	28 (7)	29
Kellgren Lawrence (KL) grade		
2	100%	Assumption
Starting WOMAC knee pain^b^	38.2 (21.8)	18
Restricted Cohort		
Inactive	32%	18
Insufficiently active	68%	18
Active	0%	18
Unrestricted Cohort		18
Inactive	16%	18
Insufficiently Active	34%	18
Active	50%	18
Annual improvement in QoL compared to inactive subjects		
Insufficiently active	0.019	10
Active	0.037	10
Annual decrease in cost compared to inactive subjects		
Insufficiently active	$351	5, 6
Active	$799	5, 6

WWE^c^ Intervention Parameters			18
*PA efficacy*			
PAG^d^ at baseline	PAG at 6 weeks	Probability	Probability of returning to background PAG each subsequent month
1^e^	1	0.05	NA
	2	0.49	0.35
	3	0.46	0.37
2^f^	1 or 2	0.34	NA
	3	0.66	0.41
3^g^	1 or 2	0.13	NA
	3	0.87	0.33
***Costs***^h^			
Start-up			$28
Per-month			$16

a Standard deviation.

b Starting pain according to the Western Ontario and McMaster Universities Osteoarthritis Index (WOMAC) pain scale (0 ​= ​best, 100 ​= ​worst).

c Walk With Ease.

d Physical activity group.

e PAG 1: Inactive (0–30 ​min of PA/week).

f PAG 2: Insufficiently active (31–179 ​min of PA/week).

g PAG 3: Active (≥180 ​min of PA/week).

h Costs are in 2020 USD and account for all costs of the Montana implementation of WWE.

##### 2.3.1.2 BMI distribution

We obtained BMI data from NHANES 2003–2006 for individuals who did and did not meet the cutoff of 180 ​min/week of MVPA [30]. For the restricted cohort, we used NHANES BMI data for those who did not meet the PA guidelines. For the unrestricted cohort, we took a weighted average of the NHANES BMI data for those who did and did not meet the PA guidelines based on the proportion of subjects in the Montana study who fell into each category.

### 2.4 Treatment characteristics

#### 2.4.1 Usual care

The usual care strategy consists of NSAIDs/physical therapy followed by intraarticular corticosteroid injections.

### 2.5 Intervention duration

Most subjects (63%) start on NSAIDs and physical therapy, while 37% have already tried NSAIDs and physical therapy and start on intraarticular corticosteroid injections as a part of the usual care strategy [12]. Subjects could stay on either regimen for up to two years.

### 2.6 Efficacy and cost

Efficacy and cost are described in detail in the Technical Appendix. Pain reduction for both the NSAIDs and physical therapy regimen and the corticosteroids regimen are stratified by baseline pain, and subjects with higher levels of pain at baseline experience greater reductions in pain due to treatment. Each subsequent month on the regimen, subjects have a probability of regressing to their baseline pain levels; this probability increases with baseline pain. Subjects incur monthly costs for NSAIDs, physical therapy, and corticosteroids as well as office visits to initiate and continue these treatments.

#### 2.6.1 Walk with ease

From 2015 to 2017, 743 persons with arthritis participated in the Montana WWE program. Participants communicated with a trainer by e-mail and walked independently. They reported their activity on a weekly basis. Persons also received financial incentives for participating in the Montana workplace wellness program, and the incentive for the WWE component was approximately $15 per month [18].

### 2.7 Intervention duration

The WWE program is designed to last six weeks, so we modeled a one-month period in which subjects could increase their activity levels. Participants could stay on the WWE regimen and incur program costs for up to two years. Subjects who discontinued the intervention would return to their initial PA level but still incur program costs because, once enrolled in the program, administrative staff communicate with subjects regardless of whether they are actively walking.

### 2.8 Efficacy

Subjects on the WWE regimen were assigned a probability in the first month of changing or staying in the same activity group, derived from the Montana study [18]. The efficacy of WWE is detailed in Table 1. Intervention efficacy was stratified by baseline pain level (see Technical Appendix for details). Of those who were inactive at baseline, five percent remained inactive after 6 weeks, 49% became insufficiently active, and 46% became active. Of those who were insufficiently active at baseline, 34% either remained insufficiently active or became inactive, and 66% became active. Lastly, of those who were active at baseline, 13% became insufficiently active or inactive, and 87% remained active. Each subsequent month, there was a 30–40% probability that subjects would regress to their initial activity level. We derived these probabilities from the Montana study 6-week and 6-month follow-up data [18].

### 2.9 Cost

The cost of the WWE program included the cost of the workbook ($12), a financial incentive ($15), and administrative costs per participant ($1). The start-up cost of WWE (incurred in the first month) is $28 (workbook, financial incentive, and administrative costs), and the cost is $16 each subsequent month (financial incentive and administrative cost).

Monthly administrative costs were calculated by determining the number of hours Montana staff dedicated to the program per month (8.5 ​h) and multiplying that by their hourly rate ($32/hour paid by the employer). This cost was then divided by the number of participants enrolled in the program each month. Administrative tasks included marketing the WWE program (to encourage participants to keep walking), answering questions from participants, and sending out weekly newsletters. All components of the start-up cost, the monthly administration cost, and the financial incentive were included in the modeled cost and represent all costs to the Montana organization.

### 2.10 Scenario analyses

In a two-way scenario analysis, we varied the WWE program cost and subsequent cycle discontinuation rate to determine how much additional funding could be invested into the program to retain participants. We added an additional $10, $50, $100, $200, $500, $700, and $1000 cost to the monthly and start-up costs of the WWE program and varied the discontinuation rate from 100% of observed to 50% of observed in increments of 10%.

### 2.11 Probabilistic sensitivity analyses

We used probabilistic sensitivity analyses (PSA) to examine how the uncertainty of model input parameters affected the outcomes. We simultaneously varied four parameters: 1) WWE program efficacy, 2) WWE program cost (start-up and per cycle; varied because monthly costs were calculated by dividing total costs per month by number of participants each month, and number of participants varied throughout the two years the WWE program was offered), 3) cost difference between PA groups, and 4) QoL difference between PA groups. The values varied and distributions used for the PSA are included in the Technical Appendix. We ran 1000 model simulations, and results of the PSA are shown using cost-effectiveness acceptability curves. These curves depict the proportion of iterations for which each strategy – usual care and WWE ​+ ​usual care – is cost-effective at a given WTP threshold.

## 3 Results

### 3.1 Base case analysis

In the base case, for the restricted cohort that is inactive or insufficiently active at baseline, QALYs over the two-year period were 1.507 for subjects who received usual care and 1.513 for those who received WWE ​+ ​usual care. Cost for subjects in the usual care regimen was $16,012 versus $16,288 for those in the WWE ​+ ​usual care regimen. Thus, the WWE regimen provides an increase in QALYs of 0.006 versus usual care at an additional cost of approximately $280 over a two-year timeframe, yielding an ICER for WWE ​+ ​usual care as compared with usual care of $47900/QALY (Table 2).

**Table 2:** Results of the cost-effectiveness of WWE in patients with knee OA.

**Inactive/insufficiently active cohort**			
Strategy	QALE^a^	Costs	ICER^b^ ($/QALY)
Usual Care	1.507	$16,012	
Walk With Ease	1.513	$16,288	$47,900
**All activity groups cohort**			
Strategy	QALE	Costs	ICER ($/QALY)
Usual Care	1.532	$15,218	
Walk With Ease	1.536	$15,526	$83,400

a Quality-adjusted life expectancy.

b Incremental cost-effectiveness ratio.

When the program was offered to the unrestricted cohort, QALYs were 1.532 for usual care and 1.536 for WWE ​+ ​usual care. The healthcare expenditure was $15,218 for usual care and $15,526 for WWE ​+ ​usual care, resulting in an ICER of $83,400/QALY.

### 3.2 Scenario analysis

**Fig. 1:**
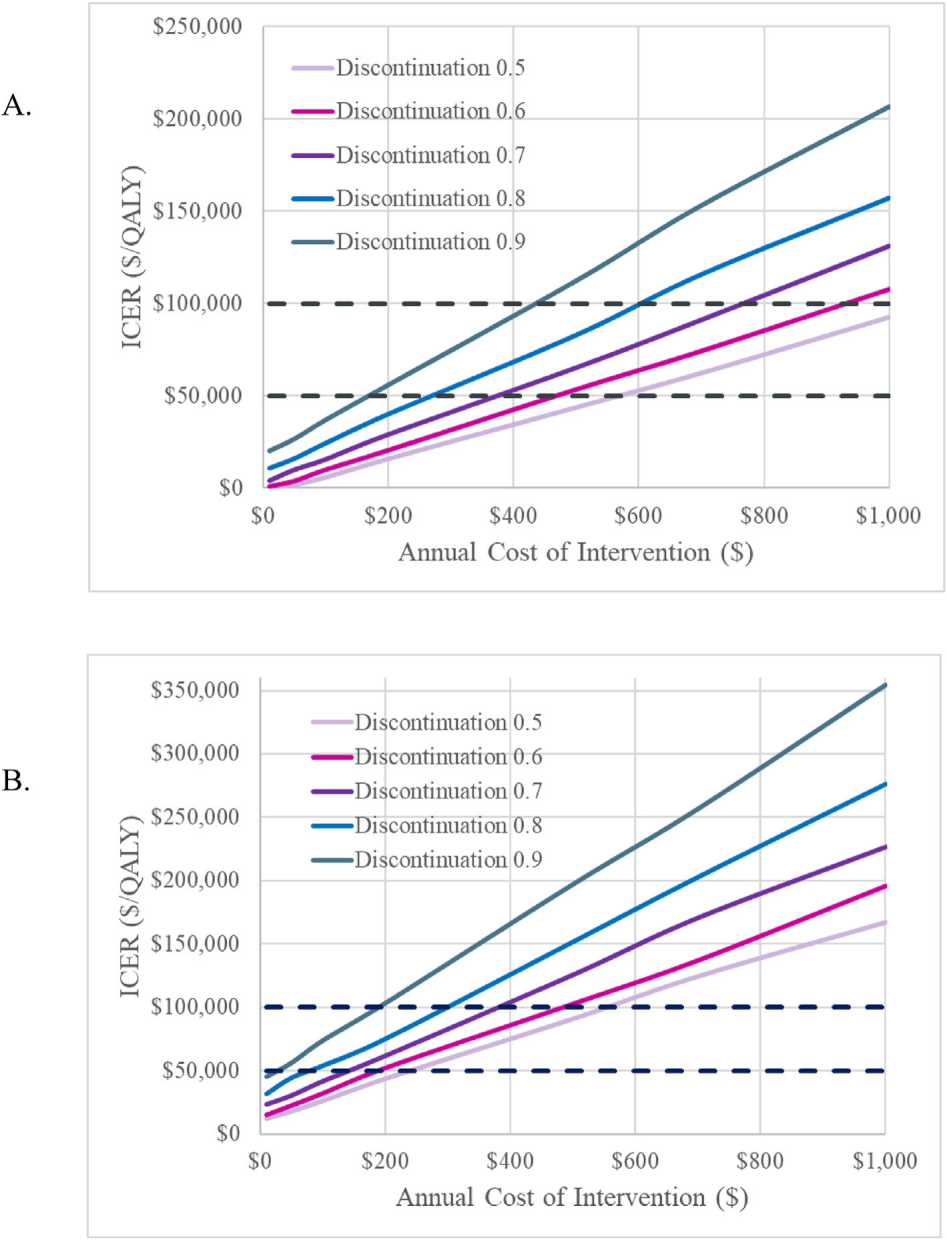
Scenario Analysis Results. Graph A shows results for the cohort consisting of inactive and insufficiently active individuals. Graph B shows results for the cohort consisting of individuals in any activity group. The distinct traces depict lower discontinuation rates than in the base case (e.g., 90% of base case, 80% of base case, etc.). At a given discontinuation rate, this figure depicts the additional amount of money (in 2020 USD) that can be invested into the program while maintaining ICERs below a given threshold. Willingness-to-pay thresholds of $50,000 and $100,000 are shown with dotted black lines.

Results of the scenario analysis are presented in Fig. 1. For the restricted cohort, scenario analyses demonstrate that when discontinuation rates are reduced to 90% of what was observed in the Montana study, an additional $36 per month ($436 per year) per person can be spent on the WWE program to maintain ICERs below $100,000/QALY. If the WTP threshold is lowered to $50,000/QALY, an additional $14 per month ($170 per year) per person can be invested into the program. When the activity discontinuation rate is reduced more drastically – to 50% of observed – an additional $1107 and $569 can be invested into the program at WTP thresholds of $100,000/QALY and $50,000/QALY, respectively.

For the unrestricted cohort, when discontinuation rates are reduced to 90% of what was observed in the Montana study, an additional $16 per month ($190 per year) per person can be spent on the WWE program to maintain ICERs below $100,000/QALY. An additional $3 per month ($30 per year) can be invested to keep ICERs below $50,000/QALY. When discontinuation is reduced to 50% of observed, an additional $46 and $20 per month ($557 and $241 per year) can be invested to maintain ICERs below $100,000/QALY and $50,000/QALY, respectively.

### 3.3 Probabilistic sensitivity analysis

**Fig. 2:**
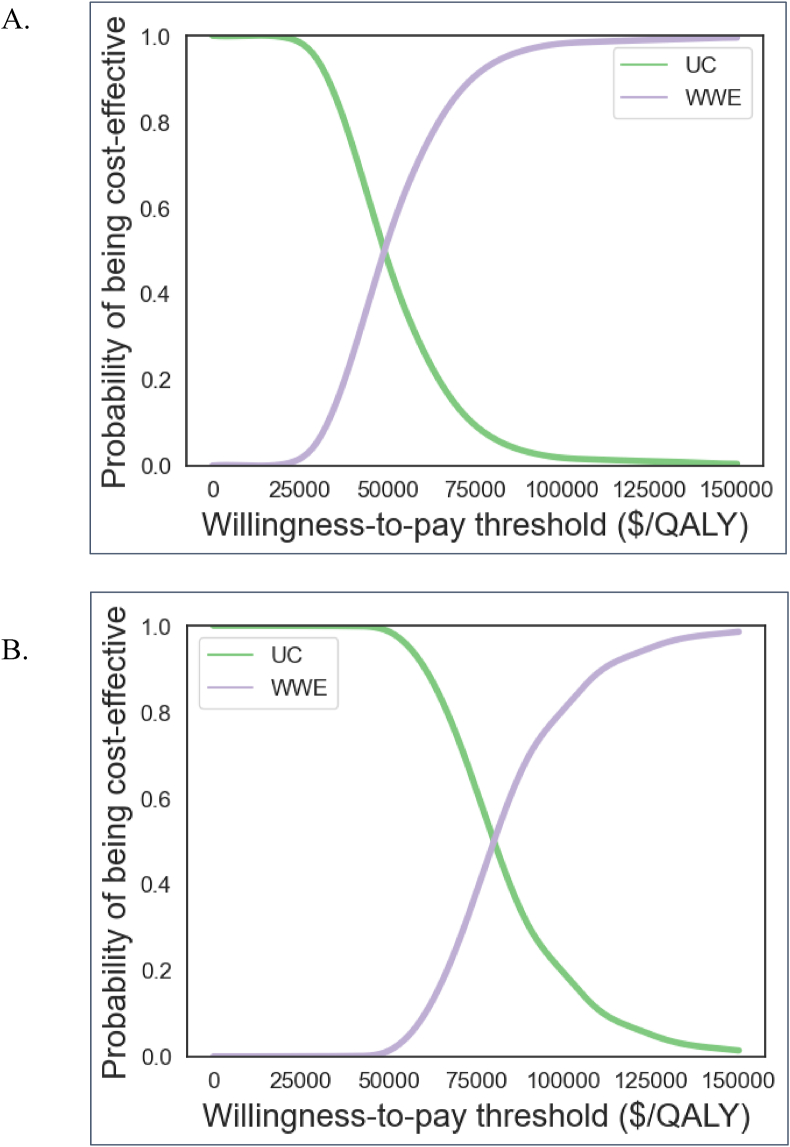
Probabilistic sensitivity analysis results. Graph A shows results for the cohort consisting of inactive and insufficiently active individuals. Graph B shows results for the cohort consisting of individuals in any activity group. The purple line depicts the probability that WWE is cost-effective at a given willingness-to-pay threshold, while the green line depicts the probability that usual care is cost-effective.

Results of the probabilistic sensitivity analysis demonstrate that at a WTP threshold of $50,000/QALY, WWE offered to inactive or insufficiently active subjects is cost-effective in 52% of iterations. 98% of iterations have an ICER below $100,000/QALY (Fig. 2A).

Without preselection for inactive and insufficiently active subjects, at a WTP threshold of $50,000/QALY, WWE ​+ ​usual care is only cost-effective in one percent of iterations. However, 81% of iterations have an ICER below $100,000/QALY (Fig. 2B).

## 4 Discussion

WWE delivers a small benefit at a low cost, resulting in favorable incremental cost effectiveness ratios and good value when delivered to participants with insufficient physical activity levels. When uncertainty in intervention efficacy, regimen cost, and QoL and cost differences between activity groups is accounted for in a probabilistic sensitivity analysis, WWE delivered to inactive/insufficiently active subjects is cost-effective in 52% of runs at a willingness-to-pay threshold of $50,000/quality-adjusted life year. When offered to subjects in all activity groups, WWE is cost-effective at a willingness-to-pay threshold of $100,000/quality-adjusted life year, but not of $50,000/quality-adjusted life year.

One major avenue for improvement of the WWE program (or similar programs that are low cost to implement) is exploring strategies to increase adherence. The small benefits of the program reflect, in part, low adherence to the WWE program (>80% of subjects either did not maintain PA improvements or did not report follow-up data at the 6-month timepoint) [18]. Previous studies have evaluated the cost-effectiveness of exercise programs for patients with knee and/or hip osteoarthritis and may inform how we can improve adherence to the WWE program. A health technology assessment comparing a group-based program consisting of two educational sessions and 24 neuromuscular exercise sessions to usual care found an ICER of $23,967 [31]. This program was much more involved than WWE and resulted in greater QALY gains (0.03 versus 0.006 for WWE) at a higher incremental cost ($719 versus $276 for WWE) over a 2-year timeframe. While both programs are cost-effective for inactive/insufficiently active participants at a WTP threshold of $50,000/QALY, WWE is a light touch intervention that could be applied at a large scale if policymakers and funders were in support and the program were easily accessible. In a cost-effectiveness analysis conducted by Fitzgerald et al. four strategies were compared: exercise only, exercise plus booster sessions, exercise plus manual therapy, and exercise plus manual therapy and booster sessions [32]. All groups received similar exercise interventions and had a total of 12 supervised therapy sessions. Those not in the booster group received all 12 sessions over the course of 9 weeks, whereas those in the booster group received eight sessions in the first 9 weeks, two sessions at 5 months, and one session at 8 and 11 months. In the cost-effectiveness analysis, strategies that contained booster sessions dominated those that did not. This suggests that booster sessions to increase adherence to exercise programs and encourage maintenance of PA levels are cost-effective. Perhaps the addition of booster sessions or more intensive follow-up with participants of the WWE program would improve program efficacy and maintenance of PA improvements over time. A United Kingdom analysis comparing a home exercise program to home exercise supplemented with a class exercise program found that home exercise ​+ ​supplemental class exercise dominated home exercise alone (lower cost and higher QALY for the former program) [33]. More recently, new technology such as mobile apps, websites, and virtual reality have been used to implement exercise programs for individuals with knee OA. A systematic review and meta-analysis of 12 technology-supported exercise programs found the interventions to have a benefit on both knee pain and quality of life [34]. Future studies may examine supplementing the WWE program with technology such as online/mobile app walking logs or community forums and assessing their cost-effectiveness.

One limitation of this analysis is that our WWE program efficacy values were derived from the Montana study, which did not have a control group (all study participants were enrolled in the WWE program). Therefore, we cannot say definitively that changes in PA are due to the WWE program as opposed to changes participants would have made without the program. However, data from the OAI suggest that PA increases are unlikely to occur in the absence of intervention [35]. Another limitation is that the Montana study cohort with arthritis included patients with any type of arthritis (rheumatoid arthritis, juvenile arthritis, osteoarthritis, etc.), whereas OAPol only models persons with knee OA. However, OA is the predominant form of arthritis [36], so we can assume that most participants in the arthritis cohort had OA. We were conservative in our assumption that the WWE program does not impact knee pain, although prior studies have suggested it may have some utility for decreasing pain, especially for those with moderate to high levels of pain at baseline. For example, in a North Carolina cohort with arthritis, joint pain, or joint stiffness who participated in the self-directed WWE program, participants had a mean improvement in pain of 8.4 points on the visual analog scale (range 0–100) [37]. Notably, the average age of the Montana study cohort was 57.5 years, while the average age of the North Carolina cohort was 64.9 years (SD 11.4). Older adults may benefit more from the WWE program, especially if they are more likely to be inactive at baseline. Montana study data were also self-reported, and the PA data were ascertained with questionnaires regarding time spent engaging in various physical activities. Self-reported physical activity data may not accurately reflect participants’ actual PA levels [38], so future studies could use objective data collected with an ActiGraph or other activity tracker. Another limitation of this study is that we did not have BMI data for the Montana cohort. We used data from NHANES 2003–2006, which may or may not be similar to that of the Montana cohort. Lastly, the mental health effects of inactivity and of exercise were not considered in this analysis, which could have resulted in an underestimation of the benefits of WWE.

The self-directed WWE program helps participants increase their PA levels over a 6-week period and is low-cost to implement. The program offers good value (ICER $47,900/QALY) if offered to inactive/insufficiently active individuals. Future research should focus on increasing participant adherence to the WWE program, perhaps through personalized approaches and the addition of low-cost technologies. Investing additional resources may increase adherence and maintain PA improvements long-term while still maintaining program cost-effectiveness. The WWE program may form the basis of a program that payers include in a treatment regimen for patients with knee osteoarthritis, especially if adherence is strengthened.

## Funding

Supported by National Institute of Arthritis and Musculoskeletal and Skin Diseases (NIAMS) grants R01 AR074290, P30 AR072577, P30 AR072580 and Centers for Disease Control and Prevention grant NU58DP006262.

## Authorship contributions

Zoe Zimmerman, Rebecca Cleveland, Aleksandra Kostic, Valia Leifer, Serena Weisner, Kelli Allen, Yvonne Golightly, Jeffrey Katz, Leigh Callahan, and Elena Losina contributed to the conception and design of the study, acquisition of data, and analysis and interpretation of data. Heather Welch and Melissa Dale contributed to the acquisition of data. Stephen Messier and David Hunter contributed to the analysis and interpretation of data. Zoe Zimmerman drafted the article, and all other authors revised it critically for important intellectual content. All authors approved the final version to be submitted.

## Declaration of competing interest

Dr. Hunter receives consulting fees from Pfizer, Lilly, Merck, Serono, TLCBio, Kolon Tissuegene, and Novartis. Dr. Losina receives consulting fees from Pfizer. Drs Katz and Losina receive grant funding from Biosplice. Serena Weisner receives funding from Flexion Therapeutics and consulting fees from the Osteoarthritis Action Alliance.
