# Supplementary material for: Walk with ease for knee osteoarthritis: A cost-effectiveness analysis
Source: Osteoarthr Cartil Open. 2023 May 10;5(3):100368. doi: 10.1016/j.ocarto.2023.100368 (PMC10206185; doi:10.1016/j.ocarto.2023.100368)
Supplement: Multimedia component — 1 [file mmc1.docx]

Contents

[1 Population Characteristics 1](#_Toc103846001)

[1.1 BMI Distribution 1](#_Toc103846002)

[1.2 KL Grade 3](#_Toc103846003)

[1.3 Background Pain 4](#_Toc103846004)

[1.4 Background Activity Level 6](#_Toc103846005)

[1.5 Background Medical Costs and Quality of Life 9](#_Toc103846006)

[1.6 Cost and QoL differences between activity groups 12](#_Toc103846007)

[1.7 Differences in comorbidity prevalence and incidence between activity groups 12](#_Toc103846008)

[2 Walk With Ease Regimen Characteristics 13](#_Toc103846009)

[2.1.1 WWE Efficacy 13](#_Toc103846010)

[2.1.2 Complications from WWE 15](#_Toc103846011)

[3 Usual Care Regimen Characteristics 15](#_Toc103846012)

[3.1 Regimen sequence 16](#_Toc103846013)

[3.2 Efficacy 16](#_Toc103846014)

[3.3 Cost 17](#_Toc103846015)

[4 Sensitivity Analyses 17](#_Toc103846016)

[4.1 Two-way deterministic sensitivity analyses 17](#_Toc103846017)

[4.2 Probabilistic sensitivity analysis 17](#_Toc103846018)

Population Characteristics

## BMI Distribution

Initial BMI distribution, stratified by gender, race, and PA group (engaging in ≥180 minutes of moderate-to-vigorous PA per week or engaging in <180 minutes per week), was derived from the National Health and Nutrition Examination Survey (NHANES) 2003-2006 (1). BMI distributions for the restricted and unrestricted cohorts are shown in tables 1a and 1b, respectively.

**Appendix Table 1a. Mean BMI – Restricted Cohort**

|  | Male | | Female | |
| --- | --- | --- | --- | --- |
|  | Mean | Standard Deviation | Mean | Standard Deviation |
| White non-Hispanic | 28.2 | 5.4 | 28.4 | 6.8 |
| White Hispanic | 28.2 | 4.7 | 29.8 | 6.2 |
| African American non-Hispanic | 28.8 | 6.2 | 31.7 | 8.0 |
| African American Hispanic | 28.2 | 4.7 | 29.8 | 6.2 |

**Appendix Table 1b. Mean BMI – Unrestricted Cohort**

|  | Male | | Female | |
| --- | --- | --- | --- | --- |
|  | Mean | Standard Deviation | Mean | Standard Deviation |
| White non-Hispanic | 28.5 | 6.0 | 28.1 | 6.7 |
| White Hispanic | 28.4 | 4.6 | 29.8 | 6.4 |
| African American non-Hispanic | 29.0 | 6.6 | 32.0 | 7.8 |
| African American Hispanic | 28.4 | 4.6 | 29.8 | 6.4 |

## Background Pain

We used the Montana study data to determine pain prevalence and pain levels at baseline. OAPol models pain using the WOMAC scale (0-100, where 100 is the worst pain) (3). 84% of subjects had pain>0 at baseline; 16% had no pain at baseline. The mean starting pain for those in pain was set to 45.4, and the mean starting pain of the cohort was 38.2 (4).

## Background Activity Level

Baseline activity group distribution was derived from the Montana study cohort with arthritis (4). Subjects were categorized into one of three groups based on their activity level: inactive (≤30 minutes of PA per week), insufficiently active (30<minutes of PA per week<180), and active (≥180 minutes of PA per week). PA data was self-reported in the Montana study (4). Participants were asked to report time spent per week engaging in each of the following activities: 1) walking for exercise, 2) swimming or aquatic exercise, 3) bicycling (including stationary exercise bike), 4) using other aerobic equipment (elliptical, rowing, or skiing machine), and 5) other aerobic exercise. They were given 5 options for quantifying time spent: 1) none, 2) less than 30 minutes, 3) 30-60 minutes, 4) 1-3 hours, and 5) 3+ hours. The sum of time spent in each activity was used to categorize participants into the various activity groups at baseline, as shown in Table 2 below. Starting PA group is stratified by age and sex.

**Appendix Table 2. Physical Activity Prevalence at Baseline for the Unrestricted Cohort**

|  | **Female** | | | **Male** | | |
| --- | --- | --- | --- | --- | --- | --- |
|  | Inactive (1) | Insufficient (2) | Active (3) | Inactive (1) | Insufficient (2) | Active (3) |
| **25-34** | 0.11 | 0.56 | 0.33 | 0.00 | 0.00 | 1.00 |
| **35-44** | 0.11 | 0.22 | 0.67 | 0.14 | 0.29 | 0.57 |
| **45-54** | 0.20 | 0.27 | 0.53 | 0.14 | 0.29 | 0.57 |
| **55-64** | 0.17 | 0.40 | 0.43 | 0.14 | 0.31 | 0.55 |
| **65-74** | 0.35 | 0.35 | 0.31 | 0.00 | 0.4 | 0.60 |
| **75-84** | 0.11 | 0.33 | 0.56 | 0.00 | 0.5 | 0.50 |
| **85+** | 0.00 | 1 | 0.00 | 0.00 | 1 | 0.00 |

For the restricted cohort (only including inactive and insufficiently active subjects), the ratio of insufficiently active to inactive subjects was the same as that in the non-restricted cohort (table 3).

**Appendix Table 3. Physical Activity Prevalence at Baseline for the Restricted Cohort**

|  | **Female** | | | **Male** | | |
| --- | --- | --- | --- | --- | --- | --- |
|  | Inactive (1) | Insufficient (2) | Active (3) | Inactive (1) | Insufficient (2) | Active (3) |
| **25-34** | 0.17 | 0.83 | 0.00 | 0.17 | 0.83 | 0.00 |
| **35-44** | 0.33 | 0.67 | 0.00 | 0.33 | 0.67 | 0.00 |
| **45-54** | 0.43 | 0.57 | 0.00 | 0.33 | 0.67 | 0.00 |
| **55-64** | 0.30 | 0.70 | 0.00 | 0.32 | 0.68 | 0.00 |
| **65-74** | 0.5 | 0.5 | 0.00 | 0.00 | 1 | 0.00 |
| **75-84** | 0.25 | 0.75 | 0.00 | 0.00 | 1 | 0.00 |
| **85+** | 0.00 | 1 | 0.00 | 0.00 | 1 | 0.00 |

The probability of changing activity groups each year was derived from the Osteoarthritis Initiative (OAI) (2) and is shown in table 4.

**Appendix Table 4. Probability of Changing Activity Group**

| **Activity Group at Baseline** | **Transition** | **Probability** |
| --- | --- | --- |
| **Activity Group 1 (Inactive)** | Increase Group | 0.00 |
|  | Remain in Same Group | 1.00 |
| **Activity Group 2 (Insufficient)** | Increase Group | 0.00 |
|  | Decrease Group | 0.08 |
|  | Remain in Same Group | 0.92 |
| **Activity Group 3 (Active)** | Decrease Group | 0.08 |
|  | Remain in Same Group | 0.92 |

## Background Medical Costs

Table 5 presents background direct medical costs, stratified by number of comorbidities, BMI, and age. These costs are derived from the 2020 CMS-HCC community model and 2017-2018 NHANES data on comorbidities (5, 6).

**Appendix Table 5. Annual Underlying Medical Costs**

| Comorbidities | Obesity | Age | Cost |
| --- | --- | --- | --- |
| 0-1 | BMI < 30 | 25-34 | $4,159 |
|  |  | 35-44 | $4,470 |
|  |  | 45-49 | $5,148 |
|  |  | 50-54 | $5,146 |
|  |  | 55-59 | $5,867 |
|  |  | 60-64 | $6,369 |
|  |  | 65-69 | $5,329 |
|  |  | 70-74 | $6,040 |
|  |  | 75-79 | $6,626 |
|  |  | 80+ | $9,120 |
|  | BMI 30 to <35 | 25-34 | $4,275 |
|  |  | 35-44 | $4,585 |
|  |  | 45-49 | $5,264 |
|  |  | 50-54 | $5,261 |
|  |  | 55-59 | $6,114 |
|  |  | 60-64 | $6,616 |
|  |  | 65-69 | $5,448 |
|  |  | 70-74 | $6,159 |
|  |  | 75-79 | $6,724 |
|  |  | 80+ | $9,216 |
| 2-3 | BMI < 30 | 25-34 | $9,968 |
|  |  | 35-44 | $10,279 |
|  |  | 45-49 | $10,917 |
|  |  | 50-54 | $10,914 |
|  |  | 55-59 | $11,521 |
|  |  | 60-64 | $12,023 |
|  |  | 65-69 | $10,817 |
|  |  | 70-74 | $11,528 |
|  |  | 75-79 | $12,114 |
|  |  | 80+ | $14,608 |
|  | BMI 30 to <35 | 25-34 | $10,084 |
|  |  | 35-44 | $10,394 |
|  |  | 45-49 | $11,032 |
|  |  | 50-54 | $11,029 |
|  |  | 55-59 | $11,767 |
|  |  | 60-64 | $12,269 |
|  |  | 65-69 | $10,936 |
|  |  | 70-74 | $11,647 |
|  |  | 75-79 | $12,212 |
|  |  | 80+ | $14,704 |
| >3 | BMI < 30 | 25-34 | $15,301 |
|  |  | 35-44 | $15,302 |
|  |  | 45-49 | $15,302 |
|  |  | 50-54 | $15,299 |
|  |  | 55-59 | $16,040 |
|  |  | 60-64 | $16,543 |
|  |  | 65-69 | $19,118 |
|  |  | 70-74 | $19,829 |
|  |  | 75-79 | $20,415 |
|  |  | 80+ | $22,910 |
|  | BMI 30 to <35 | 25-34 | $15,417 |
|  |  | 35-44 | $15,417 |
|  |  | 45-49 | $15,417 |
|  |  | 50-54 | $15,415 |
|  |  | 55-59 | $16,287 |
|  |  | 60-64 | $16,789 |
|  |  | 65-69 | $19,237 |
|  |  | 70-74 | $19,948 |
|  |  | 75-79 | $20,513 |
|  |  | 80+ | $23,005 |

## Cost and QoL differences between activity groups

Insufficiently active subjects experience lower background medical costs by $351 compared to inactive subjects, while active subjects experience lower costs by $799 (7, 8). Insufficiently active subjects have a QoL utility value of 0.019 higher than inactive subjects, while active subjects have a QoL utility value 0.037 higher. These values were derived from the OAI (2).

## Differences in comorbidity prevalence and incidence between activity groups

We model comorbidity prevalence and incidence for an inactive population. For subjects who are insufficiently active or active, we apply a relative risk reduction for both prevalence and incidence of cancer, cardiovascular disease, and diabetes mellitus.

**Appendix Table 6. Relative Risk of Comorbidity Prevalence and Incidence by Activity Level**

| Comorbidity | Insufficiently Active* | Active* |
| --- | --- | --- |
| Cancer | 0.77 | 0.76 |
| Cardiovascular Disease | 0.71 | 0.58 |
| Diabetes | 0.64 | 0.52 |
| *Risk compared to that for inactive subjects | | |

# Walk With Ease Regimen Characteristics

### WWE Efficacy

The WWE intervention had the ability to improve subjects’ PA level.

In the OAPol model, subjects are assigned a probability of moving up an activity group, moving down an activity group, or staying in the same activity group, based on their activity group at baseline. These probabilities were derived from the Montana study cohort with arthritis and are stratified by starting pain group (no to low-moderate pain (0 ≤ WOMAC pain ≤ 40) vs. moderate to severe pain (40 < WOMAC pain ≤ 100) (4). Efficacy values for the no to low-moderate pain group are shown in table 7a, and efficacy values for the moderate to severe pain group are shown in table 7b.

**Appendix Table 7a. WWE Efficacy for No to Low-Moderate Pain Groups**

| Physical Activity Group (PAG) at Baseline | PAG at 6 weeks | Probability | Probability of returning to background PAG each subsequent month |
| --- | --- | --- | --- |
| Inactive | Inactive | 0 | NA |
|  | Insufficiently active | 0.43 | 0.41 |
|  | Active | 0.57 | 0.30 |
| Insufficiently active | Inactive or insufficiently active | 0.36 | NA |
|  | Active | 0.64 | 0.38 |
| Active | Inactive or insufficiently active | 0.17 | NA |
|  | Active | 0.83 | 0.36 |

**Appendix Table 7b. WWE Efficacy for Moderate to Severe Pain Groups**

| Physical Activity Group (PAG) at Baseline | PAG at 6 weeks | Probability | Probability of returning to background PAG each subsequent month |
| --- | --- | --- | --- |
| Inactive | Inactive | 0.09 | NA |
|  | Insufficiently active | 0.53 | 0.32 |
|  | Active | 0.37 | 0.50 |
| Insufficiently active | Inactive or insufficiently active | 0.33 | NA |
|  | Active | 0.67 | 0.44 |
| Active | Inactive or insufficiently active | 0.09 | NA |
|  | Active | 0.91 | 0.31 |

### Complications from WWE

We assumed that there are no complications associated with the WWE intervention. No adverse events were reported in the Montana study (4).

# Usual Care Regimen Characteristics

The usual care for knee OA in this analysis consists of non-steroidal anti-inflammatory drugs (NSAIDs)/physical therapy (PT) followed by intra-articular corticosteroid injections. While these regimens are typically followed by total knee replacement and revision total knee replacement, this analysis is conducted over a timeframe of 2 years with subjects who start at KL-2, so we did not include these two regimens. A subject’s progression from NSAIDs/PT to corticosteroids is determined by pain and structural severity of their knee OA and treatment history, e.g., treatment success or failure in previous regimens (9). Efficacy, costs, and complications derivations have been described previously (10-12).

## Regimen sequence

63% of the cohort started on NSAIDs and 37% started on corticosteroid injections. These values were derived using the same methodology as a previous cost-effectiveness analysis of a diet an exercise program (13).

## Efficacy

The efficacy of the NSAIDs/PT regimen is detailed in table 8a below (14).

**Appendix Table 8a. Efficacy of NSAIDs/PT Regimen**

| **Pre-treatment Pain Group** | **Mean Decrement in Pain** | **SD Decrement in Pain** | **Probability of Failure in Subsequent Years** |
| --- | --- | --- | --- |
|  |  |  |  |
| Pain Group 1 | 0 | 0 | 0.24 |
| Pain Group 2 | 0 | 6.39 | 0.24 |
| Pain Group 3 | 8.90 | 15.41 | 0.24 |
| Pain Group 4 | 21.24 | 20.70 | 0.50 |
| Pain Group 5 | 36.09 | 24.71 | 0.75 |

The efficacy of the corticosteroid injections regimen is detailed in table 8b below (15).

**Appendix Table 8b. Efficacy of Corticosteroid Injections Regimen**

| **Pre-treatment Pain Group** | **Mean Decrement in Pain** | **SD Decrement in Pain** | **Probability of Failure in Subsequent Years** |
| --- | --- | --- | --- |
|  |  |  |  |
| Pain Group 1 | 0.00 | 0.00 | 0.24 |
| Pain Group 2 | 0.00 | 6.4 | 0.24 |
| Pain Group 3 | 17.4 | 19.3 | 0.24 |
| Pain Group 4 | 29.7 | 29.0 | 0.50 |
| Pain Group 5 | 44.6 | 30.5 | 0.75 |

## Cost

The monthly cost of NSAIDs/physical therapy is $50 (16). Subjects have an office visit in their first month of taking NSAIDs at a cost of $305 and an 8% probability of having a visit in subsequent months at a cost of $125 per visit (17). The monthly cost of corticosteroid injections is $40 (18). Subjects have an office visit for their injection at a cost of $122 and an 8% probability of having a visit in subsequent months at a cost of $122 per visit (17).

# Sensitivity Analyses

## Two-way deterministic sensitivity analyses

We varied the following intervention-related parameters:

1. WWE start-up and per cycle costs (adding $10, $50, $100, $200, $500, $700, and $1000 to the annual costs)
2. Subsequent cycle probability of returning to background PA level (50-100% of base case in increments of 10%)

## Probabilistic sensitivity analysis

We varied:

1. Non-intervention related
   1. Cost-differential between inactive, insufficiently active, and active subjects
   2. QoL-differential between inactive, insufficiently active, and active subjects
2. Intervention related
   1. Start-up and per cycle costs of the WWE program
      1. We specifically varied the number of subjects enrolled in the program per month. Because the cost of the program per subject was calculated as total program costs divided by the number of program participants, varying number of participants affected program costs
   2. Intervention efficacy

Table 9 summarizes the distributions used for each input parameter.

**Appendix Table 9. Probabilistic Sensitivity Analysis Distributions**

| **Gamma distributions** | | | | | | |
| --- | --- | --- | --- | --- | --- | --- |
| **Variable** | | | **Mean** | | **SD** | |
| Non-intervention related | Cost decrement for insufficiently active compared to inactive subjects | | $351 | | $171 | |
|  | Cost decrement for insufficiently active compared to inactive subjects | | $799 | | $262 | |
|  | QoL increase for insufficiently active compared to inactive subjects | | 0.019 | | 0.010 | |
|  | QoL increase for active compared to inactive subjects | | 0.037 | | 0.012 | |
| **Normal distributions** | | | | | | |
| **Variable** | |  | **Mean** | | **SD** | |
| WWE | Number of participants enrolled in WWE | | 252 | | 30 | |
| **Dirichlet distributions** | | | | | | |
| **Variable** | **Starting PAG** | | **PAG after 1 month** | **N** | | **r** |
| WWE for subjects with no-moderate pain | 1* | | 1 | 38 | | 1 |
|  |  |  | 2 | 38 | | 16 |
|  |  |  | 3 | 38 | | 21 |
|  | 2** | | 1 or 2 | 83 | | 30 |
|  |  |  | 3 | 83 | | 53 |
|  | 3† | | 1 or 2 | 119 | | 20 |
|  |  |  | 3 | 119 | | 99 |
| WWE for subjects with severe-extreme pain | 1 | | 1 | 43 | | 4 |
|  |  |  | 2 | 43 | | 23 |
|  |  |  | 3 | 43 | | 16 |
|  | 2 | | 1 or 2 | 88 | | 29 |
|  |  |  | 3 | 88 | | 59 |
|  | 3 | | 1 or 2 | 110 | | 10 |
|  |  |  | 3 | 110 | | 100 |
| *Inactive; **Insufficiently active; †Active | | | | | | |

**References**

1. National Health and Nutrition Examination Survey Data [Internet]. US Department of Health and Human Services. 2003-2006. Available from: <https://www.cdc.gov/nchs/nhanes/index.htm>.

2. Osteoarthritis Initiative (OAI). National Institutes of Health.

3. Bellamy N, Buchanan WW, Goldsmith CH, Campbell J, Stitt LW. Validation study of WOMAC: a health status instrument for measuring clinically important patient relevant outcomes to antirheumatic drug therapy in patients with osteoarthritis of the hip or knee. The Journal of rheumatology 1988;15:1833-40.

4. Silverstein RP, VanderVos M, Welch H, Long A, Kaboré CD, Hootman JM. Self-Directed Walk With Ease Workplace Wellness Program - Montana, 2015-2017. MMWR Morbidity and mortality weekly report 2018;67:1295-9.

5. Pope GC, Kautter J, Ellis RP, Ash AS, Ayanian JZ, Lezzoni LI, et al. Risk adjustment of Medicare capitation payments using the CMS-HCC model. Health care financing review 2004;25:119-41.

6. National Health and Nutrition Examination Survey Data [Internet]. US Department of Health and Human Services. 2017-2018. Available from: <https://www.cdc.gov/nchs/nhanes/index.htm>.

7. Carlson SA, Fulton JE, Pratt M, Yang Z, Adams EK. Inadequate physical activity and health care expenditures in the United States. Progress in cardiovascular diseases 2015;57:315-23.

8. Valero-Elizondo J, Salami JA, Osondu CU, Ogunmoroti O, Arrieta A, Spatz ES, et al. Economic Impact of Moderate-Vigorous Physical Activity Among Those With and Without Established Cardiovascular Disease: 2012 Medical Expenditure Panel Survey. Journal of the American Heart Association 2016;5.

9. Smith SR, Katz JN, Collins JE, Solomon DH, Jordan JM, Suter LG, et al. Cost-Effectiveness of Tramadol and Oxycodone in the Treatment of Knee Osteoarthritis. Arthritis care & research 2017;69:234-42.

10. Losina E, Paltiel AD, Weinstein AM, Yelin E, Hunter DJ, Chen SP, et al. Lifetime medical costs of knee osteoarthritis management in the United States: impact of extending indications for total knee arthroplasty. Arthritis Care Res (Hoboken) 2015;67:203-15.

11. Lenhard NK, Sullivan JK, Ross EL, Song S, Edwards RR, Hunter DJ, et al. Does screening for depressive symptoms help optimize duloxetine use in knee OA patients with moderate pain? A cost-effectiveness analysis. Arthritis care & research 2020.

12. Chen AT, Bronsther CI, Stanley EE, Paltiel AD, Sullivan JK, Collins JE, et al. The Value of Total Knee Replacement in Patients With Knee Osteoarthritis and a Body Mass Index of 40 kg/m(2) or Greater : A Cost-Effectiveness Analysis. Annals of internal medicine 2021;174:747-57.

13. Losina E, Smith KC, Paltiel AD, Collins JE, Suter LG, Hunter DJ, et al. Cost-Effectiveness of Diet and Exercise for Overweight and Obese Patients With Knee Osteoarthritis. Arthritis care & research 2019;71:855-64.

14. Clegg DO, Reda DJ, Harris CL, Klein MA, O'Dell JR, Hooper MM, et al. Glucosamine, chondroitin sulfate, and the two in combination for painful knee osteoarthritis. The New England journal of medicine 2006;354:795-808.

15. Raynauld JP, Buckland-Wright C, Ward R, Choquette D, Haraoui B, Martel-Pelletier J, et al. Safety and efficacy of long-term intraarticular steroid injections in osteoarthritis of the knee: a randomized, double-blind, placebo-controlled trial. Arthritis and rheumatism 2003;48:370-7.

16. Red Book Online. Truven Health Analytics Inc.2020.

17. Medicare Physician Fee Schedule [Internet]. Available from: <https://www.cms.gov/medicare/physician-fee-schedule/search/overview>.

18. Medicare Part B Drug Average Sales Price [Internet]2020. Available from: <https://www.cms.gov/medicare/medicare-part-b-drug-average-sales-price/2022-asp-drug-pricing-files>.
